# To compare cost effectiveness of ‘Kangaroo Ward Care’ with ‘Intermediate intensive care’ in stable very low birth weight infants (birth weight < 1100 grams): a randomized control trial

**DOI:** 10.1186/s13052-016-0274-3

**Published:** 2016-07-13

**Authors:** Deepak Sharma, Srinivas Murki, Tejo Pratap Oleti

**Affiliations:** Department of Neonatology, Fernandez Hospital, Hyderguda, Hyderabad, 500029 India

**Keywords:** Kangaroo mother care, Kangaroo ward, Kangaroo ward care’ (KWC)’, ‘Intermediate intensive care’ (IIC), Cost effectiveness, “top-down” accounting, “bottom-up” cost-accounting

## Abstract

**Background:**

To compare cost effectiveness of ‘Kangaroo Ward Care’ with ‘Intermediate Intensive Care’ in stable very low birth weight infants (birth weight < 1100 g).

**Methods:**

This is the secondary analysis of the study in which we have analysed the cost effectiveness of ‘Kangaroo ward care’ (KWC) with ‘Intermediate Intensive Care’ (IIC). In this randomized control trial 141 infants (less than 1100 g and ≤ 32 weeks at birth) were enrolled, 71 were randomized to KWC group and 70 to IIC group, once the infant reached a weight of 1150 g. Infants randomized to KWC group were shifted to the Kangaroo ward immediately after randomization. Infants randomized to IIC group were shifted to the Kangaroo ward once the infant reached 1250 g.

**Results:**

Cost incurred by the patient in both the groups from the time of randomization to hospital discharge was calculated. The hospital costs were determined by “top-down” accounting methods and out of pocket expenditure of parents from standard “bottom-up” cost-accounting methods. There was significant reduction in neonatal charges in KWC group post-randomization {41591.9 ± 21712.8 INR vs 75388.8 ± 25532.2 INR; *p* < 0.001}). The separate “top-down” and “bottom-up” cost analysis showed that there was significant reduction of hospital and parents expenditure in KWC group when compared to IIC group (*p* < 0.001). There was significant saving of around 33800 INR (USD) in the KWC group for each patient.

**Conclusion:**

Initiating early shifting to Kangaroo ward is cost effective intervention and have huge monetary implication in resource poor countries. (CTRI/2014/05/004625, retrospectively registered, Registered on: 26/05/2014).

**Clinical trial registration:**

Clinical trial registry of India CTRI/2014/05/004625 (http://ctri.nic.in/Clinicaltrials/showallp.php?mid1=7640&EncHid=&userName=CTRI/2014/05/004625) Registered on: 26/05/2014. Date of enrolment of the first participant to the trial: 13/11/2013.

## Background

Each year about 20 million infants of low birth weight (LBW) are born around the globe, which imposes a heavy burden on health care and social system in developing countries. Medical care of LBW and very low birth weight (VLBW) infants is complex, demands an expensive infrastructure and highly skilled staff. The care of LBW and VLBW is usually a very disruptive experience for families. The financial burden for managing these LBW and VLBW is huge for developing countries like India and finding a cheap and cost effective intervention is an always ongoing research in these countries [1]. KMC has shown to be the most feasible, readily available, and preferred intervention for decreasing neonatal morbidity and mortality in developing countries especially for LBW infants [2]. It is a powerful, easy-to-use method to promote the health and well-being of infants born preterm as well as full-term and in decreasing the burden on health care facilities of the developing countries [3]. Hence, we planned this secondary analysis to compare the cost effectiveness of Kangaroo ward care (KWC) versus Intermediate intensive care (IIC) in care of VLBW neonates.

## Methods

This is a secondary analysis of the randomized controlled trial which was conducted in a tertiary care nursery of the Department of Neonatology, Fernandez hospital, Hyderabad from November 2013 to August 2015. The study protocol was approved by the Institutional research board (IRB) of Fernandez hospital, Hyderabad and the study is registered at clinical trial registry, India with registration number CTRI/2014/05/004625.

Written informed consent was obtained from the parents or guardian before randomization of the infant in either of groups. They were provided with written and verbal information including on the potential risk and benefits involved on the research. One copy of research fact sheet was kept with the parents. Parents were explained about the voluntary nature of participation in the research, about non-penalization in event of non-participation and about the option of withdrawing their infant from the study at any point they wished without offering any reason. Confidentiality of the subjects was maintained.

Infants with birth weight less than 1100 g, gestation age ≤ 32 weeks, singleton, tolerating spoon or tube feeds of 150 ml/kg/day, not on intravenous fluids, breathing room air and hemodynamically stable (without any vasopressor support, normal blood pressure and maintaining temperature in incubator care with <25 % heater output) were included in the study. Infants with major malformations were excluded.

All eligible preterm infants were randomized to receive either ‘Kangaroo ward care’ (KWC) or ‘Intermediate Intensive Care’ (IIC) group once the infant reached a weight of 1150 g. Random numbers were generated by a web based computer programme (research randomizer.org). Individual group assignments were placed in a serially numbered, opaque sealed envelope that was opened only after obtaining consent from the parents.

### Intervention

#### ‘Kangaroo Ward Care’ (KWC)

Infants randomized to KWC group were shifted to the Kangaroo ward immediately after randomization. In the Kangaroo ward, care of the baby including spoon feeding, diaper change, monitoring for complications was done by the mother supervised by a trained dhai (a female attendant trained specially in taking care of neonates and skilled in doing daily care activities of the neonates like spoon feeding, ensuring breast feeding in term infants, changing diapers, clothing, early detection of neonatal complications and advising mother on baby care). The infants were kept in skin-to-skin contact (KMC), firmly attached to the mother’s chest with a cloth binder during KMC sessions. Front open gowns were made available for the mothers and privacy was provided to them for their use. Comfortable chairs and beds were provided for the mothers for practicing KMC. The duration in which the infant was not in skin to skin contact, the infant was wrapped properly with clothes, cap, and socks and placed in a well cushioned baby swaddlers. Mothers were taught to identify hypothermia and cold stress.

#### ‘Intermediate Intensive Care’ (IIC)

Infants randomized to IIC group were cared for in the intermediate care area of the NICU. Infants were cared under incubator/warmer in servo control mode for thermoregulation. Mothers were encouraged to visit the baby as many times as possible and were encouraged for skin to skin contact in the intermediate care unit for as long as possible. When the infants were not in skin to skin contact with the mother, it was in the incubator/warmer. All the baby care activities were done by the neonatal nurses. When the infant reached 1250 g, it was shifted to the kangaroo ward and subsequently, the baby care was similar to that in the KWC group.

In both the groups, mothers were trained to do KMC for as many hours per day as possible ensuring a minimum of six hours per day. If infant was on tube feeds, feeding was done by a trained nurse or dhai. Infants were discharged home at a minimum weight of 1400 g and gaining weight of ≥ 10gm/day on 3 consecutive days. Mothers were encouraged to continue skin to skin contact at home as long as the baby was tolerating it.

Feeding in both the groups was expressed breast milk (EBM) given with a paladi (a traditional spoon used for feeding neonates in India) at 2 hourly intervals. EBM was supplemented with human milk fortifier (HMF) for as long as the infant was on gavage or paladi feeds. When on direct breastfeeds, human milk fortifier was replaced with calcium, phosphorus, multivitamins and iron supplements. When expressed breast milk was not available a preterm formula was used. Supplements were used as per the unit protocol.

The data was collected in a predesigned proforma. The data included baseline variables like birth weight, weight at enrollment, discharge weight, gestation at birth, at enrollment and at discharge, sex, antenatal steroids (complete, partial, nil, multiple courses), mode of delivery (vaginal/cesarean), one minute and five minute Apgar, nutrition (partial /total parental nutrition), postnatal age at full feeds and also neonatal morbidities like blood culture positive sepsis, Necrotizing enterocolitis (NEC) and Stage [4], Patent ductus arteriosus (PDA) (medical or surgical management) [5], ROP and staging [6], IVH/PVL and grading [7, 8] and the cost variable after the randomization.

### Cost analysis

Cost incurred by the patients in both the groups was from the time of randomization to hospital discharge. The hospital costs were determined by “top-down” accounting methods and included consultant charges, specialist charges, duty doctor charges, nurse’s charges, supporting staff charges, intervention charges, laboratory investigation charges and procedural charges if any. The out of pocket expenditure of parents was calculated from standard “bottom-up” cost-accounting methods and included the charges paid by the parents from the time of randomization till discharge.

### Statistics

Comparisons between study groups for discrete variables was performed with the chi-square or Fisher’s exact test. Continuous variables were compared by means of Student’s *t* test or nonparametric tests, when appropriate. It was an intention to treat analysis and all patients were analyzed according to the group to which they were allocated, regardless of compliance with treatment or contamination of the intervention. The foreign exchange rate used in the analysis was 66 Indian rupees = 1 USD (2015).

## Results

**Fig. 1:**
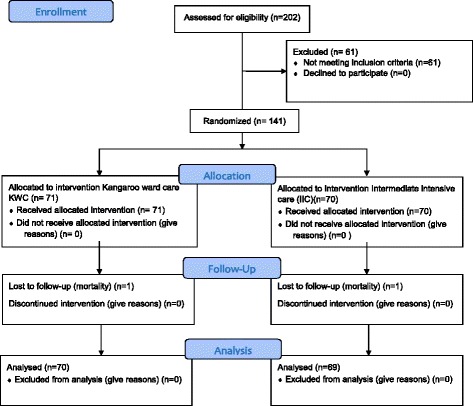
Flow diagram of the study population

During the study period 202 infants were assessed for enrolment, of which 61 were excluded. Of the excluded infants, ten were pair of twins, two were sets of triplets, two had oxygen dependency at the time of enrollment, ten infants left against medical advice before enrollment and 23 were neonatal deaths (Fig. 1). So from November 2013 to August 2015, total of 141 infants were enrolled.

### Comparison of baseline variables between the two groups (Table 1)

**Table 1:** Baseline characteristics of the study population

Variables	‘Kangaroo ward care (KWC)’ (71)	‘Intermediate intensive care’ (70)
Mean Birth weight (gram)	953.66 (127.9)	984.86 (113.3)
Mean Length (cm)	36.37 (1.8)	36.00 (2.5)
Mean Head circumference (cm)	26.04 (1.2)	25.95 (1.5)
Gestation (Mean ± SD)	29.27 (1.8)	29.13 (1.9)
Male sex n (%)	33 (46.5)	41 (58.6)
IUGR *n* (%)	21 (29.6)	19 (27.1)
C section *n*, (%)	63 (88.7)	66 (94.3)
Antenatal Steroids *n* (%)	68 (95.8)	69 (98.6)
EBM at Randomization *n* (%)	66 (93)	69 (98.6)
5 min Apgar (Median, IQR)	8 (7–8)	8 (7–8)
Ventilated *n* (%)	19 (26.8)	13 (18.6)
Culture positive Sepsis *n* (%)	15 (21.1)	19 (27.1)
NEC *n* (%)	9 (12.7)	8 (11.4)
PDA *n* (%)	21 (29.6)	13 (18.6)
Mean Duration of TPN (days)	8.5 (4.8)	9.4 (4.2)
Mean Time to reach full feeds (days)	10.1 (5.9)	10.8 (4.3)

*NEC* Necrotizing enterocolitis, *PDA* patent ductus arteriosus, *TPN* total parenteral nutrition, *EBM* expressed breast milk, *IQR* inter quartile range, *IUGR* intrauterine growth restriction, *C* section- caesarean section

Of the 141 infants enrolled, 71 were randomized to KWC group and 70 to IIC group. The baseline variables including birth weight, length and head circumference were comparable between the two groups (*p* > 0.05).

### Comparison of interventions in the two groups (Table 2)

**Table 2:** Intervention table of the study population

Variable	‘Kangaroo ward care’ (KWC) *n* = 71	‘Intermediate intensive care’ (IIC) *n* = 70	*P* value
Age at |randomization (days)	29.13 (13.8)	26.7 (11.6)	0.3
Gestation at randomization (weeks)	33.3 (1.8)	32.9 (1.9)	0.2
Weight at randomization (gram)	1152.5 (12.0)	1152.6 (13.4)	1
Length at randomization (cm)	38.2 (1.5)	38.3 (1.6)	0.6
Head circumference at randomization (cm)	27.4 (1)	26.9 (1.2)	0.01

*NICU* neonatal intensive care unit, *KMC* Kangaroo mother care, *EBM* expressed breast milk

The postnatal age, gestational age, weight, length at randomization was similar in both the groups (*p* > 0.05). There was significant difference in head circumference at the time of randomization (27.4 ± 1 cm vs 26.9 ± 1.2 cm, *p* = 0.01).

The combined cost effective analysis (“top-down” and “bottom-up”) showed significant reduction in neonatal charges in KWC group (41591.9 ± 21712.8 INR) when compared to IIC group (75388.8 ± 25532.2 INR; *p* < 0.001). The separate “top-down” and “bottom-up” cost analysis showed significant reduction of hospital and parents expenditure in KWC group (*p* < 0.001) (Table 3). There was a saving of nearly 33800 INR (512 USD) for each patient in the KWC group.

**Table 3:** Table showing cost difference between the two groups using “top down” and “bottom up” method

Variable	‘Kangaroo ward care’ (KWC)	‘Intermediate intensive care’ (IIC) *n* = 70	*P* value
“Top down” Hospital charges	24955.1 ± 13027.7	45233.3 ± 15319.4	<0.001
“Bottom-up” Cost incurred by parents	16636.7 ± 8685.1	30155.5 ± 10212.9	<0.001

## Discussion

In this secondary analysis we report the cost effectiveness of “Kangaroo ward care” in stable VLBW infants in comparison to “Intermediate intensive care”. Early shifting of infants to KMC ward where baby care was by the mother led to significant reduction of cost to the parents using “bottom-up” method and hospital cost using “top- down” method. This cost difference can be explained by significant reduction in both post randomization NICU stay (KWC 1.7 ± 3.4 days vs 7.2 ± 3.0 days IIC) and post randomization hospital stay in KWC group (KWC 14.8 ± 5.2 days vs 17.0 ± 5.8 days). In this study the cost was calculated from randomization to ‘discharge from hospital’, post randomization stay and total number of days during which neonate received IIC and KWC were same. The results this study show that KWC is a cheap and easily doable intervention and can save nearly 512 USD per infant in a developing country setting where financial burden to family from hospital costs is a big problem.

Cattaneo et al. evaluated the cost efficiency of KMC when compared to conventional methods of care (CMC). The cost variables included two types of running costs: salaries and other items (food for mothers and babies, laundry and linen, drugs and other medical supplies, X-ray and laboratory, fuel and electricity and maintenance of equipment). The cost of salaries was calculated by estimating the proportion of staff time spent on KMC and CMC and multiplying it by the number of months worked during the study by different categories of health workers; the other costs by keeping accurate monthly records. The authors showed that KMC was cheaper than conventional methods of care (CMC) in terms of salaries (US$ 11,788 vs US$ 29,888) and other running costs (US$ 7501 vs US$ 9876) [9]. The savings from KMC in comparison to CMC are approximately 40 % of the total cost as in the trial from Cattaneo et al and nearly 50 % as in our trial.

Jannati et al. conduced a retrospective cost effective analysis of KMC and CMC. In the study infants randomized to CMC group were nursed either under servo controlled radiant warmers or in a cradle under hot lamps in the NICU. The total cost was calculated and the cost of each procedure was determined by standard “bottom-up” cost-accounting method. The resource utilization was recorded for each procedure, and the cost of each item was estimated on the basis of the hospital acquisition cost for the item. All other hospital costs were determined by “top-down” accounting methods based on hospital’s annual accounting report. They showed the mean cost of hospitalization per individual infant for KMC was 3539.47$, whereas for CMC group it was 2907.27$ [10]. The cost savings of nearly 630$ per patient in the study by Janani et al is close to the savings reported by us in this study.

In a previous study from our institute comparing KMC with Conventional care in stable very low birth weight infants, post randomization , infants in the KMC group had an average of 11.5 days lesser stay in the NICU. This shorter stay in the NICU resulted in a mean cost saving of nearly US $ 500 (25,000 INR) for each patient enrolled in the KMC group compared with that enrolled in the CMC group [11]. This results are comparable to present study as in both study cost bared by the parents has been taken in to account. Both the study groups (KWC and IIC) in this trial are similar to the KMC arm of our previous trail. However, the mean weight at randomization for the infants in the KWC group in this trail is lower than the mean weight at randomization for the infants in the KMC arm of our previous trial. Thus early initiation of KWC as in this study may further add to the cost reduction for stable preterm infants. Thus combing the results of this study and our previous study very ealry initiation of KWC in stable preterm infants, may on an average save nearly 1000$ to each patient in comparison to conventional care.

### Strength of study

1. Relatively large sample size and prospectively collected data.
2. Calculated cost saving for the  parent in KMC group which is the need of the parents.
3. Cost analysis done using “top down” and “bottom up” accounting method.
4. Well established Kangaroo care in KMC ward and NICU.

## Conclusion

Kangaroo mother care is a cheap and cost effective intervention in both developed and developing countries. The mere implementation of KMC in the care of low birth weight infants in developing countries will lead to significant monetary for the parents and also decongestion of NICU, reduced role of nursing care and involvement of mother in baby care.

## Abbreviations

CPAP, continuous positive airway pressure; EBM, expressed breast milk; ELBW, extremely low birth weight; FCC, Family centred care; INR, Indian Rupees; IVH, intraventricular haemorrhage; IIC, intermediate intensive care; KMC, Kangaroo mother care; KWC, Kangaroo ward care; LBW, low birth weight; NEC, Necrotizing enterocolitis; PDA, patent ductus arteriosus; PVL, periventricular leukomalacia; ROP, retinopathy of prematurity; SSC, Skin to skin contact; SD, standard deviation; VLBW, very low birth weight
